# Modern Trends in Public Health
*An abridged version of an address to the Bristol Medico-Chirurgical Society given on Wednesday 14th March, 1956.


**Published:** 1956-10

**Authors:** R. C. Wofinden

**Affiliations:** M.O.H., City and County of Bristol


### MODERN TRENDS IN PUBLIC HEALTH*

BY

R. C. WOFINDEN, M.D., D.P.H.,

*M.O.H., City and County of Bristol*

I am deeply honoured to be asked to address this learned society. I do so with some trepidation for I find that we Medical Officers of Health have appeared before you at very infrequent intervals. Indeed, the last occasion was forty-seven years ago and the first, and only other occasion, was in 1900 when Dr. D. S. Davies gave his Presidential Address entitled "Some Modern Aspects of Preventive Medicine". You will see that my title "Modern Trends in Public Health" is almost the same but progress in the past half-century has been so great and so rapid that I cannot hope to do more than touch on some of the more recent developments in the public health field.

### GENERAL PRACTITIONER–PUBLIC HEALTH–HOSPITAL RELATIONSHIPS UNDER THE NATIONAL HEALTH SERVICE ACT, 1946.

When the provisions of the National Health Service Act were first read by M.O's.H., and Local Authority representatives they occasioned great despondency. The Local Authorities cleaned up the environment in the "Sanitary Era" and then developed first-class facilities for personal health through their maternity and child welfare and school health services. After providing infectious disease hospitals and sanatoria they were on the way to building up a sound general hospital service and there was a chance for the real integration of prevention, treatment and after-care. Indeed, the 1930's during which much of this progress was made has often been referred to as "the Augustan age of public health". Then, in a short Act of Parliament containing no more than eighty sections, the carefully bonded cords of integration and co-ordination were cut asunder. The difficulties, indeed the folly, of splitting the health service into the three divisions of hospitals, general practice and public health, are well known to us all. Looking back over the past eight years it is astonishing that developments have proceeded so smoothly and the degree of co-ordination achieved, despite the administrative boundary lines speaks highly for the goodwill and co-operation on the part of all the workers in the different branches. It is indeed something to be thankful for that not all the boundary lines have become frontiers.

If all branches had been placed under one administration, however, I have no doubt that more rapid and greater progress could have been made. A much more closely integrated service would have been produced and planning could have been on rational lines unencumbered by the eternal battle of rates versus taxes. I would remind you that in 1955–56 the gross expenditure of the Exchequer on the National Health Service was estimated at £477 $\times$ $10^6$. Sixty four per cent. of this money was to be spent on hospital and specialist services, 28 per cent. on general practitioner services and only 4 per cent. on Local Health Authority Services.

Naturally, when we are ill we all want to have the best possible treatment and for some conditions this can only be obtained in hospital. But those of us who work in the public health services would like to see, if we are going to have a fixed overall budget, some redeployment of expenditure with more spent in trying to lessen the need for expensive hospital treatment. I am sure that general practitioners too are beginning to realize that their future vested interest lies in prevention. There are indications that many conditions treated in hospital today might with economy be

*An abridged version of an address to the Bristol Medico-Chirurgical Society given on Wednesday 14th March, 1956.

catered for in the home. This will only be done by developing a first-class partnership between the practitioner and the local health authority field workers—the health visitor, the midwife, the home nurse and home help and with the help of the hospital consultant or specialist. Let me give one illustration. In 1953 nearly two-thirds of mothers in this country were confined (and many of them were normal cases) in hospital where costs are in the region of £20 a week. Even with midwives, a general practitioner, a flying squad if need be, and some home help assistance, a home confinement is estimated to cost only about one-third of this amount. To look at the same problem in another way, a distinguished hospital administrator estimated that one maternity bed less will release enough money for three mental hospital beds, four mental deficiency beds or three geriatric beds.

In the field of sick children too there are signs appearing of the flight from the hospital. We read of hospital units being established to house mother as well as the sick baby in order to avoid psychological damage to the child by separation from its mother. A Dutchman has gone so far as to say that it is "psychological murder" to nurse sick children in hospital. Not all of us would subscribe wholly to the Dutchman's thesis but apart from psychological considerations the Medical Officer of Health for Rotherham has shown the possible life-saving effect of nursing sick children at home instead of in hospital and I have no doubt we shall see further developments in this field.

No doubt, too, you will have seen recent reports of some 400 hernia cases sent home from hospital almost immediately after operation only a very small proportion of which needed readmission.

Developments such as these might not save a lot of money directly but they would shorten waiting lists and get people fitter more quickly. It would mean the further development of the Local Health Authority home services but this would be far less costly than building, equipping and staffing more hospital beds.

Hospitals are very necessary—but on psychological grounds they should be places to avoid entering as far as possible and places to get out of as soon as possible.

You will see from what I have said that I believe we need to orientate our health services around the home rather than have them hospital centred. The home, with its family unit, is a more natural setting for the treatment of sickness than the feverish and highly artificial atmosphere of the hospital. Moreover, it is the common meeting-ground of prevention and treatment. Most people spend at least two-thirds of their daily lives in and around the home—their health influenced by the people, the environment and the social circumstances surrounding them. This is the setting so well known to the general practitioner and health visitor. Here are generated many of the stresses and strains of everyday life which affect the health of the individual. I believe it is one of the duties of the public health services to give every assistance to the general practitioner in performing his work in the home.

Moreover, strengthened home care services and closer co-operation with the hospital consultant are needed for the after-care or rehabilitation of those discharged from hospitals. Professor Ferguson of Glasgow has shown by a follow-up of cases sent home from a medical ward how few of those discharged had been able to take their place as healthy members of society. Professor Querido of Amsterdam has produced similar findings for a whole range of patients discharged "cured" or "relieved" from hospital. Hospitals unfortunately often have to treat the end-results of pathological processes but we should do all we can to render what necessarily must often be patchwork as effective as possible. Here and there we see the beginnings of a more efficient after-care service—notably in Cardiff where specialized health visitors assist the general practitioner in the hospital after-care of diabetics, and peptic ulcer and asthma cases.

### MODERN EPIDEMIOLOGY

I suppose there are many people who still regard epidemiology as the study of the origins, development and distribution of epidemic diseases. Undoubtedly, when epidemics of pestilential disease were prevalent their systematic study in the mass was the preoccupation of our epidemiologists who, for the most part, were to be found in public health departments.

Today, most of the epidemic infectious diseases are under control, and epidemiology is concerned increasingly with the study of the origins and causes of non-infective conditions; indeed, with the whole range of human disease. It has been defined as the "science of the mass phenomena of disease and disability". Morris has pointed out that "The clinician deals with cases. The epidemiologist deals with cases in their population. He may start with a population and seek out the cases in it; or start with cases and refer them back to the population, or what can be taken to represent a population." We are beginning to realize that many diseases may have their origins not so much in the physical environment as in our social milieu—i.e., in our way of life, within the home, or in the community or at our work. As our social habits and customs change with time so does the pattern of disease. Undoubtedly, also, one must try to take into account constitutional factors in any such aetiological studies.

Morris has described seven different ways of looking at epidemiological data:

(*a*) The historical approach—the study of diseases over a period of time to see whether they are declining or increasing and he instances the current trend of mortality in middle age in Western countries.

(*b*) Community diagnosis—the identification of vulnerable groups in the community, e.g. in regard to foetal and infant loss.

(*c*) To estimate the individual's chances of suffering or not suffering from certain diseases or disabilities and he shows how this can be done through a life-table approach.

(*d*) Operational research—the study of how community health and welfare services are working. My pilot investigation of multiplicity of home visiting is a good example of this approach, also my old-age studies.

(*e*) For completing the clinical picture. Morris goes on to show that statistics of "coronary thrombosis" based on hospital returns alone give only about a half of the total cases which probably occur—the other half being dealt with by general practitioners or the coroner.

(*f*) The Identification of Syndromes—As Ryle has shown from mortality statistics for "peptic ulcer" there are probably two different conditions to be studied with probable different causes.

(*g*) Clues to Causes—e.g. by a study of mortality from certain diseases in groups of the population subjected to varying work loads; the possible relationship of bronchitis and other lung diseases with urbanization; the possible relationship between smoking and lung cancer and so on.

Much of the time of the personnel working in public health departments is spent in working in special groups in the population—the pre-school child, the school child, the aged, or the handicapped and they are in an excellent position to carry out such epidemiological studies. Some health departments are in fact carrying out such studies either alone or on behalf of central research bodies. Undoubtedly, at present, these aetiological studies can only give broad indications of where to look further for causes without pin-pointing specific aetiological factors. Moreover, to be really effective these studies must be based on accurate and comprehensive diagnostic data and this often can only be obtained from general medical practitioners or hospital specialists.

Hitherto, only certain of the infectious or industrial diseases have been compulsorily notifiable. If we are to make further epidemiological progress it will be necessary to extend and improve the technique of notification. I am not suggesting that all disease processes should be compulsorily notifiable but with thought and careful planning I have little doubt that a good case could be made for the notification of certain of the killing diseases which are showing an increasing incidence.

There is already a precedent, for the Ministry have approved the plans of one Local Health Authority to make coronary artery disease a notifiable condition.

### THE PREVENTION OF MENTAL ILL HEALTH

I believe that another big public health task that we have to tackle in the next few decades is to reduce, if we can, the burden of mental ill health. Today we have a crisis on our hands with nearly half our hospital beds filled with cases of mental disorder and mental deficiency and with a chronic and worsening shortage of mental nurses. The Government and the profession are pre-occupied in how to continue bolstering up this salvage work. I believe the time has come when we must direct our thoughts to the possibilities of preventing mental ill health at least that portion of it concerned with emotional mal-adjustment.

Local Health Authorities have been assigned this task under Section 28 of the National Health Service Act under which we have the duty to prevent "illness" and "illness" includes "mental illness". Ferguson Roger has written:

> "mental and neurotic illnesses are so prevalent that we are never likely to solve the vast problem which they present by increasing our resources for treatment."

Obviously no local authority can hope to fulfil this task without the co-operation of the hospital consultant and specialist and the general practitioner. But first it will be necessary to fight a long and hard battle of educating lay persons in positions of authority and the general public for we shall be able to move no faster than public opinion allows. At present, as soon as one starts talking about providing preventive mental health services, fears, prejudices and feelings of guilt appear to be aroused.

There was a time when it was hoped that the child guidance clinic, by giving guidance at an early stage to parents of maladjusted children, would prevent much adult mental ill-health. I cannot judge for myself how far the child guidance clinic has succeeded in this task but I would like to quote Elizabeth Irvine to you—a lady of considerable personal experience in this field:

> "It became gradually clear that, however much child guidance facilities are multiplied, however great the extension of early treatment, even this expanded therapeutic service would touch only the fringe of the mental hygiene problem. The only possible answer is to develop a true preventive service."

How, then, is such a service, or the beginnings of such a service to be provided?

I believe, with Elizabeth Irvine, that it is necessary to give "skilled and sensitive first aid at the earliest opportunity" and that this can be most readily achieved by the Maternity and Child Welfare Departments and the general practitioners. "There is only one service which has routine contact with nearly all mothers and babies—the Maternity and Child Welfare service, and the professional groups to whom the mother brings her problems are the medical officers and the health visitors in this service, together with the general practitioners." To enable public health doctors and nurses to do this job would necessitate some modification of existing training programmes; it would also mean training and employing more of them to facilitate smaller case loads. Much can be done, however, as is being shown in the London County Council, by in-service training of existing staff. Such schemes as I suggest are being tried in other countries, e.g. in the Vancouver Health Department where they are referred to as "anticipatory guidance" in child health centres.

We have made a small start in this city by the employment of two psychiatric social workers who are working in the Maternity and Child Welfare department and it is my earnest hope that from these small beginnings we shall go on to develop a service of real preventive value.

### HEALTH CENTRES

I have referred to the disadvantages of split administration in the health services. Undoubtedly the architects of the National Health Service must have hoped that "Humpty Dumpty" could be put together again by housing together in health centres the workers in the preventive and therapeutic fields. I could not let this occasion pass without some reference to the William Budd Health Centre in Bristol—the first centre to be built by a Local Health Authority. It has been open now for three and a half years and in my opinion, and in the opinion of all who work there and of the hundreds of visitors (many of them from overseas) who have studied it, the Centre has attained considerable success. It is a pity that some of its greatest critics have never visited the Centre. It is one of the few places I know where professional and lay staff from all three branches of the health service are working together under one roof. Misunderstandings between general practitioners and public health workers have been removed and all are working together as a team with the aim of improving the health and welfare of the people living in the locality. It is an experimental venture and since its inception additional new aids have been provided for the general medical practitioner—e.g. the nutrition clinics and the services of a psychiatric social worker.

It was at the William Budd Health Centre that we made our first (and very successful) attempt to streamline the City's ante-natal and post-natal services. Many general practitioners are now seeing their patients in the City's clinics in association with the domicilary midwives, and with the help of one of the Board's obstetric consultants. They have been relieved of many of their non-productive tasks and the Local Authority has been able to continue its health educational activities amongst a very important group of the population. The fact that so many expectant mothers can now receive all, or almost all, their care in one place is a step in the right direction. No one would claim that we have achieved a fully streamlined service but I know that many other towns would welcome a similar organization.

Some of us would like to see radiological and clinical pathology facilities provided for *all* the doctors working in the centre area. Radiological and pathology consultants would need to supervise the service. One of the greatest advantages of such a scheme would be the *ready* accessibility of the consultants to the general practitioners. I know that it is now possible for general practitioners to refer cases for investigation to hospital radiology and pathology departments and to discuss their cases with the consultant. But many general practitioners are far removed from, and haven't the time to visit, hospitals; moreover, there would be the essential difference in a health centre that the practitioner was working on his "home ground".

The William Budd Centre has been criticized on the grounds of its expense. It is serving a population of nearly 20,000 for Local Health Authority maternity and child welfare and school health purposes, and there are some 12,000 patients at risk on the doctors' lists. I think you might agree therefore that a lot of preventive and therapeutic service is being provided for the £10,000 per annum it costs to run.

There have been a number of reports in recent years criticizing general medical practice in England and Wales and stressing the need to up-grade it. To my mind the provision of health centres, where the doctor can be relieved of non-productive tasks and where he can be given the tools to do this job, would be the ideal way of doing it. If I am right in saying that the vested interest of the general practitioner is no longer in disease but in health we must give him the opportunity to teach health. Each consultation provides such an opportunity but it cannot be grasped unless we provide him with the necessary time in which to do it. Such time can be found in health centres. The alternative seems to me to be the training and employment of many more doctors so that case loads could be reduced.

### PROBLEM FAMILIES

In recent years health departments have shown an increasing interest in the reclamation of problem families. Their characteristics are well-known to you—the squalor of the home, the instability of marriage and family relationships, the repeated running into debt, child neglect, alcoholism, and the discord with their neighbours and the community in which they live.

They have been thrown into relief whenever they have been rehoused from slum quarters on the new housing estates where they fall short of prevailing social standards.

Undoubtedly each problem family presents a unique family problem which has to be tackled as such. But they have certain characteristics in common—mental subnormality coupled with instability of character of one or both parents. Many appear to be ineducable by any present-day methods. They are a problem to the many statutory and voluntary workers from whom they receive help. General medical practitioners, hospital specialists and public health doctors and nurses know how difficult it is to get the parents to co-operate in treatment of either themselves or their children.

Until comparatively recently it was common practice to prosecute the parents for child neglect (the penalty for which was usually a prison sentence) and to take the children into official care. Now, however, public opinion is no longer so hostile and there seems to be a genuine desire to help them back to normal life. Partly from economic necessity (i.e. to reduce the expense of looking after children in children's homes) and partly from the powerful influence of Bowlby's report on "Maternal Care and Mental Health" there is now a widespread acceptance of the view that whenever possible the family should be kept together as a unit.

In the first place, some attempt is being made to reduce the number of visitors to their homes; this helps to cut out the often fragmentary and sometimes conflicting advice which was given previously. Co-ordination is being achieved through case conferences at which it is usual for representatives of statutory and voluntary organizations working in the area to be present. At such meetings their problems can be discussed in detail, responsibility for future care assigned to one worker and all other agencies learn which worker must be kept informed. Often the responsible home worker is the health visitor, sometimes the children's officer or the welfare officer of the Welfare Department. At such conferences I have been impressed repeatedly by the variety of health problems—particularly in the mental sphere but also of physical ill health—which become manifest. There is undoubtedly a need to spot these problems at a much earlier stage and to try to put them right.

Family Service Units are doing a good job of work on their behalf; sometimes their workers are more acceptable to the problem family than the statutory workers but not always so. The keynote of their work is to forge friendly relationships and to help the family to build up self-reliance and independence. The secret of much of their success lies in the small case loads they accept—thus ensuring that sufficient time is spent with each family.

You must all have been impressed with the poor health of many of these mothers who so often bear a large number of children very rapidly. For them a spell of three months at a recuperation centre such as Brentwood is often invaluable, not only to allow the mother to recoup her health but also to let her regain her self-respect and to receive training in mothercraft and housewifery. A few Local Authorities have their own recuperation centres and we hope in due course to provide one in Bristol.

Many of us believe that these families ought to be spread thinly throughout the community in reconditioned properties rather than concentrating them on new housing estates. This is what we are doing in Bristol and we are finding that in such units and with intensive family case work one can often prevent the family from breaking up or reconstitute the family which has already broken up.

It is clear that our efforts on their behalf are still patchy and relatively unco-ordinated. There is a great need to find out more about the root cause of problem families and I feel sure that there is need for much help from our colleagues who specialize in mental health.

### FUTURE DEVELOPMENTS IN ENVIRONMENTAL HEALTH

Although the nineteenth century was referred to, in the public health world, as the environmental or "sanitary era" we should realize that notable advances have been made in the improvement of our environment in the last fifty years, particularly in housing and slum clearance. But much remains to be done, for new environmental problems are constantly arising out of our changing mode of life and work. We have scarce begun to tackle the problem of providing a satisfactory working environment for many of our factory workers and for our shop and office workers. Rapid developments are taking place in the food industry whereby more and more of our natural foodstuffs are being processed in one way or another—"sophisticated" is the modern word—in order to render them more attractive to the customer. This sophistication, involving as it does, chemical additions to foods, or their subjection to various rays may not be entirely free from risk and at present goes on almost unregulated. I must confine myself to only one development in the environmental health services which we shall see well under way during the coming decade—the provision of clean air.

### ATMOSPHERIC POLLUTION

For many years The Smoke Abatement Society waged a continuous but unsuccessful battle for the abatement of atmospheric pollution. The evils of pollution such as waste of fuel, damage to agriculture, buildings and clothing and to health have been realized for a long time. In recent years two disasters to the public health have arisen from atmospheric pollution coupled with abnormal weather conditions—the Meuse Valley episode in 1930 when sixty people died and many cattle were killed, and the Donora disaster in the U.S.A., when twenty people died and half the population of 14,000 was made ill. Both of these disasters were far from home and evinced little local interest.

Then, in December 1952, we had the London "smog" disaster which in a single week killed 4,000 persons, and caused an untold amount of illness. The cause was well understood. Some 200–500 ft. above the ground was a stationary layer of warm air below which was trapped a shallow layer of cold air polluted from tens of thousands of chimneys—domestic and factory ("temperature inversion"). The deaths were due to respiratory disorders and concomitant cardiac failure. All age groups were affected but more particularly those over the age of forty-five years. But it was not just a case of old people dying who were going to die shortly anyhow. The death curve rose with age, following a course parallel with the normal curve which showed that many people died a good deal before their time. It is strongly suspected that sulphur dioxide was the cause of much of this trouble. In the London smog disaster the atmospheric $SO_2$ content was between 14–50 times normal.

It is reasonable to suppose that the underlying cause of the high mortality in a smoke fog must operate continuously although in a lesser degree, on the health of urban communities. It may well swell the ranks of the 30,000 deaths a year from chronic bronchitis. Coupled with these bad effects on the respiratory tract is the deprivation of town dwellers from their quota of sunlight. It is well known, for example, that in the winter months the light in the centre of a city may be only one-half the quantity on the outskirts.

Evidence is also accumulating that coal smoke may be among the factors contributing to the increase in bronchial carcinoma. Coal smoke is known to contain benzpyrene which is carcinogenetic. Moreover, the incidence of lung cancer is higher in towns, especially the smokier towns, than in rural areas and it may be that coal and tobacco smoke have a reinforcing effect in this regard.

A Clean Air Bill is now before Parliament and there is little doubt that many of its provisions relating to pollution from domestic and industrial chimneys will be enforceable by the Local Authorities' Inspectorate. Progress may be impeded if the Government allow too many escape clauses in the Bill, by the shortage of smokeless fuel and by the shortage of skilled inspectors. Given the will to succeed and continuing education of the public we shall see considerable progress in this next ten years.

Tonight, I have tried to give you an idea of some of the present-day trends in the public health world. Time has not permitted consideration of other interesting developments—in services for the aged and the handicapped, of the progress being made in health education or in the prevention of tuberculosis.

I hope, Mr. President, that after listening to me tonight some of you will be able to agree with what Sir Geoffrey Vickers said in his address at the opening of the session of University College Hospital Medical School in 1954:

> ". . . when I hear people say that public health has ceased to be a live and expanding field—and even doctors have been heard to say this—I wonder whether their idea of preventive medicine is as modern as it might be."

Some of you may have been thinking that our range of activities is too wide—even to the extent of encroaching on territory which you might not consider traditional or orthodox public health ground.

I would remind you, however, that the duties of the Medical Officer of Health as laid down in the Sanitary Officers (Outside London) Regulations 1935 are that:

> "The Medical Officer of Health shall inform himself, as far as practicable, respecting all matters affecting or likely to affect the public health in the district, and be prepared to advise the Local Authority on any such matter."

With this "brief" behind him and the provisions of Section 28 of the National Health Service Act opening up unlimited possibilities of developing preventive health services I think the future of public health is by no means so gloomy as some have believed. Ira Hiscock has said that "public health is a purchasable commodity" and I look forward to the day when governments will be more willing to purchase it.

